# Neonatal enteral feeding tubes as loci for colonisation by members of the *Enterobacteriaceae*

**DOI:** 10.1186/1471-2334-9-146

**Published:** 2009-09-01

**Authors:** Edward Hurrell, Eva Kucerova, Michael Loughlin, Juncal Caubilla-Barron, Anthony Hilton, Richard Armstrong, Craig Smith, Judith Grant, Shiu Shoo, Stephen Forsythe

**Affiliations:** 1 School of Science and Technology, Nottingham Trent University, Clifton Lane, Nottingham, NG11 8NS, UK; 2 Life and Health Sciences, Aston University, Aston Triangle, Birmingham, B4 7ET, UK; 3 Nottingham City Hospital, Nottingham, NG5 1PB, UK; 4 Queens Medical Centre, Nottingham, NG7 2UH, UK

## Abstract

**Background:**

The objective of this study was to determine whether neonatal nasogastric enteral feeding tubes are colonised by the opportunistic pathogen *Cronobacter *spp. (*Enterobacter sakazakii*) and other *Enterobacteriaceae*, and whether their presence was influenced by the feeding regime.

**Methods:**

One hundred and twenty-nine tubes were collected from two neonatal intensive care units (NICU). A questionnaire on feeding regime was completed with each sample. *Enterobacteriaceae *present in the tubes were identified using conventional and molecular methods, and their antibiograms determined.

**Results:**

The neonates were fed breast milk (16%), fortified breast milk (28%), ready to feed formula (20%), reconstituted powdered infant formula (PIF, 6%), or a mixture of these (21%). Eight percent of tubes were received from neonates who were 'nil by mouth'. Organisms were isolated from 76% of enteral feeding tubes as a biofilm (up to 10^7 ^cfu/tube from neonates fed fortified breast milk and reconstituted PIF) and in the residual lumen liquid (up to 10^7 ^*Enterobacteriaceae *cfu/ml, average volume 250 μl). The most common isolates were *Enterobacter cancerogenus *(41%), *Serratia marcescens *(36%), *E. hormaechei *(33%), *Escherichia coli *(29%), *Klebsiella pneumoniae *(25%), *Raoultella terrigena *(10%), and *S. liquefaciens *(12%). Other organisms isolated included *C. sakazakii *(2%),*Yersinia enterocolitica *(1%),*Citrobacter freundii *(1%), *E. vulneris *(1%), *Pseudomonas fluorescens *(1%), and *P. luteola *(1%). The enteral feeding tubes were in place between < 6 h (22%) to > 48 h (13%). All the *S. marcescens *isolates from the enteral feeding tubes were resistant to amoxicillin and co-amoxiclav. Of additional importance was that a quarter of *E. hormaechei *isolates were resistant to the 3^rd ^generation cephalosporins ceftazidime and cefotaxime. During the period of the study, *K. pneumoniae *and *S. marcescens *caused infections in the two NICUs.

**Conclusion:**

This study shows that neonatal enteral feeding tubes, irrespective of feeding regime, act as loci for the bacterial attachment and multiplication of numerous opportunistic pathogens within the *Enterobacteriaceae *family. Subsequently, these organisms will enter the stomach as a bolus with each feed. Therefore, enteral feeding tubes are an important risk factor to consider with respect to neonatal infections.

## Background

Recently, considerable attention has been directed at the microbiological safety of PIF [1,2]. This has primarily been due neonatal infections by *C. sakazakii *and *Salmonella*, which were associated with contaminated PIF [3-6]. These products are not sterile, but are expected to comply with international microbiological standards [7]. Other *Enterobacteriaceae *which have been isolated from PIF include *Enterobacter cloacae*, *Klebsiella pneumoniae, K. oxytoca, E. hormaechei*,*Citrobacter freundii*, and *E. coli *[8,9]. The FAO/WHO [1,2] categorised these organisms as *'causality plausible, but not yet demonstrated' *with respect to their potential to cause neonatal illness through the ingestion of reconstituted PIF. Although these organisms are opportunistic pathogens, there have been no confirmed outbreaks in NICUs attributed to their presence in contaminated PIF. This in part may be related to misidentification and delays in investigation. For example, reinvestigation of a *C. sakazakii *outbreak on a NICU revealed the organisms were *E. hormaechei *[9]. In another NICU outbreak, the powdered infant formula was not analysed until after the last neonatal case and the original batch of infant formula would no longer have been available [3]. The FAO/WHO [1] proposed that the risk of bacterial infection from powdered infant formula could be reduced by reconstitution with water > 70°C, minimising the time between reconstitution and feeding (< 2 h), and by not storing reconstituted feed at ambient temperature. These recommendations are reiterated by WHO [10], and various regulatory bodies [11-13]. However, there was no consideration that the nasogastric enteral feeding tube may act as a site for bacterial colonisation as a biofilm. The tube will be between ambient (outer portion) and body temperature (inner portion), with regular additions of nutrients from the infant feed and in-place over sufficient time periods for bacterial multiplication. Previously, nasogastric feeding tubes in a nursing home have been shown to be a reservoir for *E. coli *and *Klebsiella *with extended spectrum β-lactamases (ESBL) [14]. Berthelot *et al*. [15] proposed a role of enteral feeding in the colonisation and infection of premature infants by *K. oxytoca*. In recent years there has been a rise in incidence of neonatal infections due to *Enterobacteriaceae*, and they are the predominant causative agents in NICU outbreaks [16-18]. *Klebsiella *spp. infections outnumber staphylococci infections, and *Serratia *spp. are the third most common causative pathogen [16]. Pathogenic strains of *E. coli *are one of the leading causes of neonatal meningitis and sepsis [19]. Neonates may be particularly prone to Gram negative infections as their innate immune cells have low responses to lipopolysaccharide (part of the Gram negative cell wall structure) and macrophage response [20].

Prior to weaning, the infant intestinal flora is influenced by the feeding regime [21]. The initial intestinal flora of infants who are breast fed are dominated by lactic acid bacteria and bifidobacteria, whereas the intestinal flora of formula fed infants is more diverse and dominated by the *Enterobacteriaceae *and *Bacteroides *spp. [22]. However, this is a generalisation, as in practice neonates may receive a mixed nutrient source regime for short periods according to their nutritional needs. This may include the use of thickeners to reduce reflux, and these details may not be sufficiently recorded for later analysis. Few studies have considered the neonatal nasogastric enteral feeding tube in NICUs acting as a site of bacterial colonisation, and any influence of the feeding regime. Mehall et al. [23] detected *Staphylococcus epidermidis*, *S. aureus*, *Enterococcus faecalis*, *E. cloacae *and *K. pneumoniae *at > 10^3 ^cfu/ml in 71/125 enteral tubes from infants > 4 months, and that necrotizing enterocolitis developed in 7 formula fed infants with tubes containing > 10^5 ^Gram negative bacteria/ml. This group also reported the isolation of methicillin-resistant *S. aureus *from infant enteral feeding tubes [24]. Therefore collating information on hospital feeding regimes, and microbial analysis of feeding tubes will considerably improve our knowledge and understanding of potential risk factors to neonates linked to enteral feeding. This study is important to identify locations of bacterial multiplication which might be of risk to neonates, especially in NICUs.

## Results

### Neonate feeding regime

A total of 129 nasogastric enteral feeding tubes were collected from two NICUs; 25 and 104 respectively. The neonates' age range was from < 1 wk to greater than 4 wk, with the major group (42%) being > 4 wk (See additional file 1). Four specific feeding regimes were identified; 'breast milk', 'fortified breast milk', 'ready to feed formula', and 'reconstituted PIF'. Additionally, a number of neonates were receiving more than one type of feed. These are described as receiving a 'mixed feeding regime'. This latter category is a heterogeneous population. For example, some neonates received breast milk and fortified breast milk, whereas others received breast milk and reconstituted PIF. A thickener was added to feeds to reduce reflux for neonates receiving fortified breast milk, ready to feed formula, reconstituted PIF, and mixed feed. Ten tubes were received from neonates that were 'nil by mouth' (see additional file 1). The frequency of feeding was primarily every 2 h for breast milk, whereas there were equal numbers of every 2 h and 3 h for those receiving ready to feed formula (see additional file 1). Eight neonates were fed ready to feed formula, reconstituted PIF, and mixed feed continuously. The enteral feeding tubes had been in place for various time periods; ranging from < 6 h (22%) to > 48 h (13%) (see additional file 1). The gastric pH was measured prior to feeding, and was between 1.5 and 6. The average pH ranged from 2.5 to 4.3 for breast milk and reconstituted PIF fed neonates, respectively (see additional file 1).

### Microbiological analysis of enteral feeding tubes

#### Bacterial counts on tubes

*Enterobacteriaceae *were isolated from the majority (76%) of samples, and from all feeding regimes (see additional file 2). The lowest frequency of isolation was 52% of the tubes from breast milk fed neonates, whereas the others ranged from 78 to 88% for mixed feeding regime and reconstituted PIF (see additional file 2).

**Figure 1:**
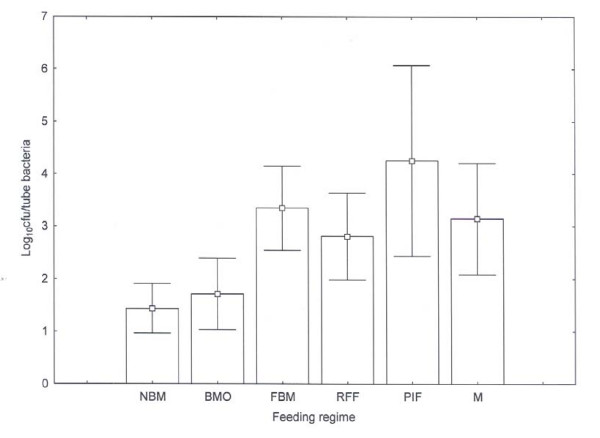
***Enterobacteriaceae *counts from biofilm material isolated from nasogastric enteral feeding tubes of neonates on various feeding regimes**. Error bars indicate 95% confidence intervals. NBM = Nil by mouth (n = 10). BMO = Breast milk only (n = 17), FBM = fortified breast milk (n = 27), RFF = ready to feed formula (n = 21), PIF = Powdered infant formula (n = 8), M = mixed feeding regime (n = 20).

The dataset for NICU 2 (n = 104) was chosen for detailed statistical analysis due to the larger number of samples, and the 'nil by mouth' cohort was regarded as a control group for the feeding regimes. Feeding regime had a significant effect on the *Enterobacteriaceae *counts (F = 3.90, P < 0.001). The lowest values obtained were 'nil by mouth' and 'breast milk' only with an average values ca. 1.4 log_10 _cfu/tube (Fig. 1). The maximum 'nil by mouth' was 2.7 log_10 _cfu/tube which was less than the average for the remaining groups. The maximum for the breast milk cohort was 5.3 log_10 _cfu/tube. This value was considerable higher than the other neonates in the cohort, and could have been influenced by the exceptionally high pH (6.0) of this one sample. Fischer's protected least significant difference *post-hoc *tests suggested that 'fortified breast milk', 'ready to feed formula', 'reconstituted PIF' and 'mixed formula' all gave significantly greater counts than 'nil by mouth' regime. Hence, 'breast milk' and 'ready to feed' gave bacterial counts similar to those on the 'nil by mouth' regime. Statistical analysis showed that although there was a significant number of younger babies (< 1 wk) in this group, there was no statistical significant difference in colonisation between age groups (Analysis of variance 1-way F = 0.99, P > 0.05). Similarly, within the fortified breast milk group there was no significant effect of age on colonisation (Analysis of variance 1-way F = 0.89, P > 0.050). Hence, although there were some differences in age profiles within treatment groups and an overall effect of age, there was no evidence from our data that age effects colonization within a feeding group. It is accepted that the numbers within each group are small for these comparisons and it is possible that there were confounding problems of age with feeding regime. Within the mixed feeding regime group, analysis of the various feeding regimes suggested that those fed with breast milk and fortified breast milk, and PIF and breast milk, gave significantly higher counts than nil by mouth (F = 3.19, P < 0.05), but not the ready to feed formula and breast milk group. There was no difference in bacterial counts when a thickener was added to the feed.

**Figure 2:**
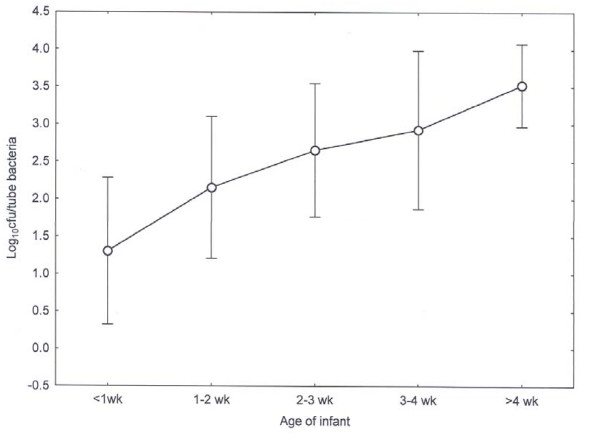
***Enterobacteriaceae *counts from biofilm material on nasogastric enteral feeding tube according to age of neonate**. Error bars indicate 95% confidence intervals.

The effect of age of the infant on mean bacterial counts is shown in Fig. 2. There was a significant effect of age on bacterial counts when data were pooled (F = 4.49, P < 0.001) indicating a progressive increase in numbers with increasing age from 2 wk onwards. There was a significant effect of length of time the tube was in place when data were pooled (F = 6.91, P < 0.001). Compared with data at < 6 h, those at 6-12 h, 18- < 24 h, 24-48 h, and > 48 h, all had significantly greater bacterial counts, with maximum counts recorded at 48 h. When data are pooled, there was a positive but weak correlation between *Enterobacteriaceae *numbers and pH (r = 0.24, P < 0.05). However r^2 ^suggests only 5.8% of the variance in bacterial numbers can be accounted for by pH. A one-way Anova comparing pH with age showed there was no significant difference in average pH between infant age classes. There were no significant effects of age within the 'nil by mouth' group (Analysis of variance 1-way F = 10.00 P > 0.05).

#### Bacterial counts in residual liquids

During laboratory analysis of the feeding tubes, it was noted that there was residual liquid present. The average volume of residual liquid was 250 μl, and ranged between 30 and 400 μl. The average viable count was 10^7 ^cfu/ml, and ranged between < 10^2 ^to 10^8 ^cfu/ml. Therefore, the number of *Enterobacteriaceae *present in the residual liquid per tube was up to 10^7 ^cfu. This is the potential number of *Enterobacteriaceae *that would have entered the neonatal stomach with the next feed, if the tube had not been removed.

#### Bacterial species on tubes and in the residual liquids

**Figure 3:**
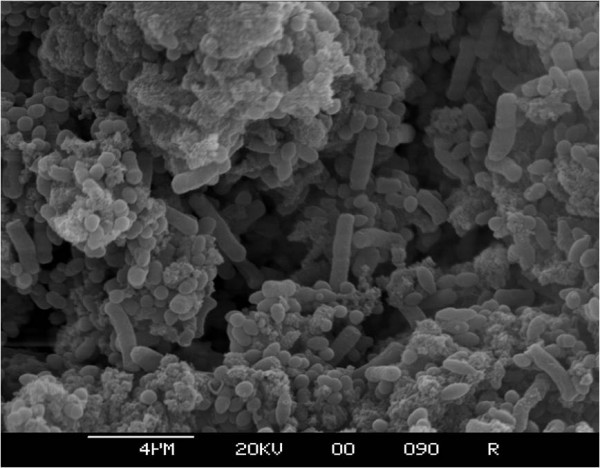
**Electron microscopy of enteral feeding tube inner wall from neonate fed breast milk and ready to feed formula**. Bar indicates 4 μm size marker.

**Figure 4:**
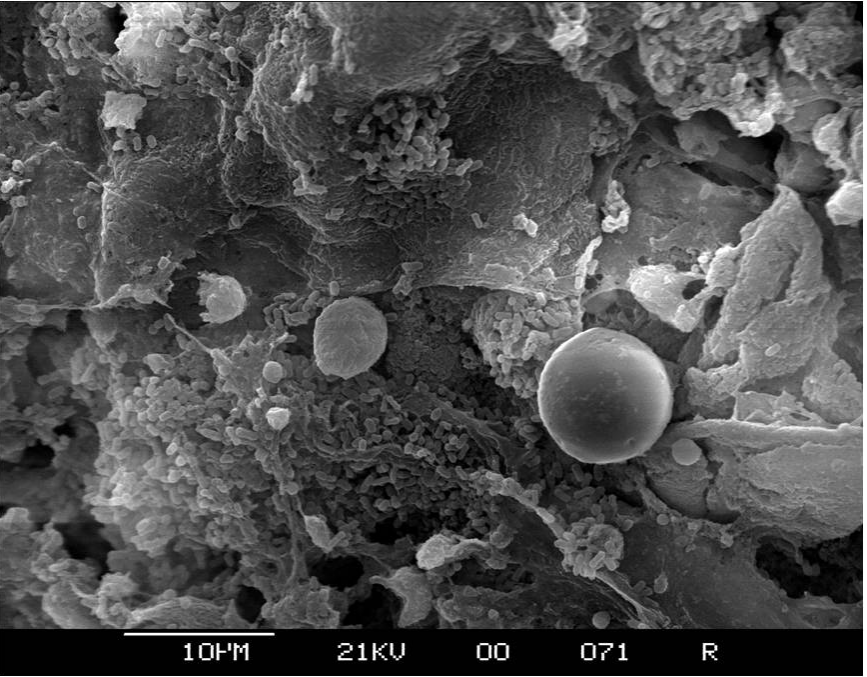
**Electron microscopy of enteral feeding tube inner wall from neonate fed breast milk and reconstituted PIF with added thickener**. Bar indicates 10 μm size marker.

The same *Enterobacteriaceae *species were isolated from both the residual liquids and the biofilms. The *Enterobacteriaceae *isolated were primarily *E. cancerogenus *(41%), *S. marcescens *(36%), *E. hormaechei *(33%), *E. coli *(29%), *K. pneumoniae *(25%), and *R. terrigena *(22%) (see additional file 2). Other organisms isolated less frequently included *C. sakazakii *from breast milk and ready to feed formula groups, and a single isolate of *Y. enterocolitica *from the reconstituted PIF group. *E. cancerogenus*, *S. marcescens*, and *E. hormaechei *and were isolated from all feeding regimes, including the 'nil by mouth' cohort (see additional file 2). The *E. hormaechei *and *E. cancerogenus *(identified by16S rDNA sequence analysis) were presumptively identified as *E. cloaceae *and *K. oxytoca*, respectively, by phenotypic profiling. Non-*Enterobacteriaceae *which were isolated from VRBGA included *P. fluorescens*, *P. luteola *and *Chromobacterium violaceum*. Electron microscopy of enteral feeding tube inner wall revealed that a dense, and morphologically diverse flora was present (Fig 3 and 4). This included a variety of short and long rod-shaped bacteria; some with tapering ends (Fig. 3). Yeast size cells were also visible (Fig. 4). Preliminary experiments with direct plating of enteral tube material on non-selective agar isolated staphylococci, lactic acid bacteria and *Candida albicans *(data not shown). Since these were not the focus of the study, they were not investigated further. There was no significant difference in the proportion of samples positive for *Enterobacteriaceae *between the feeding regimes (chi-square = 7.82, 5DF, P > 0.05). The distribution of bacterial species was different in tubes from 'nil by mouth' samples compared with all other regimes added together (chi-square = 16.28, 7DF P < 0.05). After removing the 'nil by mouth' samples from the statistical analysis, there were highly significant differences in the distribution of isolates between feeding regimes (chi-square = 94.95, 28DF, P < 0.001). Comparing each feeding regime with each of the others, showed that the breast milk and mixed feeding regimes were the only two giving similar distribution of isolates (chi-square = 9.72, 7DF, P > 0.05). Each of the other feeding regimes has a unique distribution of bacterial isolates.

#### Antibiotic resistance of isolated *Enterobacteriaceae*

All *Enterobacteriaceae *isolates were susceptible to gentamicin, ciprofloxacin and meropenem. The majority of strains were resistant to amoxicillin. The antibiograms for the remaining antibiotics are summarised in additional file 3. All *S. marcescens *isolates were resistant to amoxicillin and amoxicillin-clavulanic acid. Of note is the high frequency of resistance to ceftazidime (21% strains) and cefotaxime (23% strains) in *E. hormaechei*. Three of these strains contained ESBL. Four of the 37 *E. coli *strains were also resistant to ceftazidime and cefotaxime.

## Discussion and Conclusion

In this study, a total of 129 nasogastric enteral feeding tubes with details of the neonates' feeding regime were obtained from 2 NICUs. The neonates were receiving a variety of feeds including expressed breast milk, reconstituted PIF, and sterile ready to feed formula. In addition tubes were received from infants that were 'nil by mouth'. The ages of the neonates varied with the feeding regime. Those on breast milk were predominantly < 1 wk, whereas those on fortified breast milk were > 4 wk (see additional file 1). Neonates receiving ready to feed formula were 1 to > 4 wk in age, whereas the majority of those on reconstituted powdered infant formula were > 4 wk in age. The frequency of feeding was primarily (54%) every 2 h, especially for those on breast milk, and secondly (24%) every 3 h. Eight neonates (6%) were fed continuously. This later practise is prone to temperature abuse, and has been linked with outbreaks of *C. sakazakii *in USA and France [3,25].

*Enterobacteriaceae *were isolated from the lumen and the inner wall of most (75%) enteral feeding tubes; see additional file 2. The organisms were initially identified using biochemical profiles and thereafter 16S rDNA sequence analysis as the later is more accurate [9,26]. The *Enterobacteriaceae *isolated were primarily *E. coli*, *E. cancerogenus*, *E. hormaechei*, *K. pneumoniae*, *R. terrigena*, and *S. marcescens*. These are well recognised opportunistic pathogens causing various gastrointestinal and respiratory diseases. Other organisms isolated included *C. sakazakii*, *Y. enterocolitica, E. vulneris*, and *Pseudomonas *spp. There were some differences in the flora between the feeding regimes, the reasons for which are currently unclear. The flora isolated from our neonatal samples is similar to that of Mehall et al. [24] who reported the isolation of *E. cloacae*, *K. pneumoniae*, *S. maltophila *and *P. aeruginosa *from enteral tubes of infants aged > 4 months. Other organisms present included Gram positive organisms such as staphylococci, and lactic acid bacteria, as well as *Candida albicans*. This fungus was also isolated in the study by Mehall et al. [24].

The antibiograms for the *Enterobacteriaceae *isolated are shown in additional file 3. Trimethoprim, ampicillin and co-amoxiclav are commonly used for minor infections in adults. Piptazobactam, amikacin, ceftazidime and cefotaxime are antibiotics that could be prescribed for empirical treatment of serious sepsis in infants on a neonatal intensive care unit. Of note is the high frequency of resistance by the *E. hormaechei *to the 3^rd ^generation cephalosporins ceftazidime and cefotaxime. ESBL were detected in 3 of these strains. The antibiotic resistance patterns of the remaining strains could be due to derepressed chromosomal AMPC β-lactamase production [27]. As already proposed [28], it is plausible that the empiric use of antimicrobial agents selects for clones of EBSL organisms such as *S. marcescens *and *K. pneumoniae*. Although no link was established with feeding tube isolates, it is notable that these two species were also responsible for neonatal infections in both NICUs during our study. Resistance to these antibiotics would not be recognised until 24 - 48 h of culturing for the causative agent, during which time an ineffective antibiotic may have been used to treat the ill neonate. This delay in effective treatment could have serious consequences.

The 'nil by mouth' samples received during the study were treated as negative controls for the feeding regime comparison. They demonstrated that sterilisation of the outer tube surface effectively removed any oral-pharynx flora contamination. Therefore the organisms detected were deemed to originate from the inside of the enteral feed tube. It is probable that the few organisms isolated from these tubes originated from the throat by tracking along the outside of the tube into the stomach, or were residual organisms from before feeding stopped. Due to respect for strict patient confidentiality, all neonates were anonymous and hence we have no knowledge regarding the feeding regime of neonates prior to the sampling period. This unfortunately restricts our interpretation of data obtained for neonates that were 'nil by mouth' at the time of sample collection. Nevertheless, only low numbers of *Enterobacteriaceae *(< 3 log_10 _cfu/tube) were recovered from these samples (Fig. 1).

The *Enterobacteriaceae *were isolated from biofilms inside enteral feeding tubes of neonates who received only breast milk, but the numbers were lower than other feeding regimes (Fig. 1). Breast milk is not sterile, but does contain antibacterial agents such as maternal antibodies, lactoferrin, and lysozyme. Additionally the standard practice at the 2 NICUs was for expressed breast milk to be kept at 2-4°C for no more than 48 h and therefore very little bacterial growth could have occurred prior to feeding. Feeding tubes from neonates being fed fortified breast milk contained higher numbers of *Enterobacteriaceae *than unfortified breast milk; 3.6 log_10 _cfu/tube compared with 1.4 log_10 _cfu/tube respectively (Fig. 1). Human milk fortifiers may enable bacterial growth by providing free iron which is otherwise unavailable due to chelation in unfortified breast milk [29]. Another factor which may have affected the bacterial counts is that neonates fed fortified breast milk were older than those fed breast milk (see additional file 1). Some of the enteral tube flora could have been due to reflux of small intestinal contents into the stomach. Since older neonates will have a more established intestinal flora, increased bacterial numbers would be recovered from enteral tubes in the stomach. The 37 fortified breast milk tube samples were treated as one cohort since to our knowledge only one source of human milk fortifier was in use.

An unexpected result was the recovery of *Enterobacteriaceae *biofilms in enteral feeding tubes from 81% of neonates receiving sterile ready to feed formula. These products are sterilised inside glass jars by the manufacturer and have tamper-proof lids which would indicate any bacterial growth before use. These feeds were used directly from the sterile jar, and were not kept open for any length of time at temperatures enabling bacteria from extrinsic contamination to multiply. An alternative source of the enteral tube flora was the throat due to gastroesophageal reflux. This is common in preterm neonates, occurring 3-5 times per hour [30,31]. It occurs when the lower oesophageal sphincter relaxes, and this may increase the exposure of the feeding tube to the throat flora.

The highest *Enterobacteriaceae *biofilm levels were from enteral feeding tubes of neonates receiving reconstituted PIF; average 4.2 log_10 _cfu/tube. We have no knowledge regarding the range of PIF products being used on the wards, but it is reasonable to assume that various products had been prescribed by the neonatologists. However requesting further nutritional information was not permissible with respect to patient confidentiality. Therefore all neonates receiving reconstituted PIF were considered as one cohort. The same *Enterobacteriaceae *species were isolated as per other feeding regimes; *E. coli*, *E. cancerogenus*, *R. terrigena*, and *S. liquifaciens*; see additional file 2. Other *Enterobacteriaceae *isolated were *Y. enterocolitica*, *K. ozaena *and *C. violaceum*. Whether these *Enterobacteriaceae *originated from the powdered formula or reflux from the gastrointestinal tract is uncertain as no bacteriological analysis of the powdered formula was undertaken. Nevertheless the PIF were reconstituted at room temperature and therefore were not subject to hot water (> 70°C) to reduce the number of any intrinsic bacteria as recommended by the FAO/WHO [1,2]. Since, unlike human breast milk, there are no antibacterial agents in PIF any contaminating bacteria would be able to multiply in the formula while the tube was in place for up to 48 h.

As the bacterial biofilms age, the *Enterobacteriaceae *will break off in clumps. These clumps will inoculate any fresh feed in the tube lumen leading to further bacterial multiplication, and will subsequently enter the neonate stomach. Although the adult stomach is normally highly acidic, and kills the majority of ingested bacteria, this is not true for the neonate. The gastric pH was 2.5 (breast milk) and 3.5 to 4.3 for the remaining feeding regimes; see additional file 1. Edelson-Mammel et al. [32] have shown the acid-sensitivity of *C. sakazakii*. In their study of 12 strains, the viability decreased by 1 - 3.5 log cycles at pH 3.5 over a 5 h period. Koutsoumanis and Sofos [33] reported that the viable counts of *E. coli *O157:H7, *Listeria monocytogenes*, and *Salmonella *Typhimurium decreased by 2, 4 and 7 log cycles respectively when subjected to the same conditions. It is plausible that gastric juices were sometimes present in the enteral feeding tube which could reduce the biofilm formation. This may account for the absence of *Enterobacteriaceae *in tubes from neonates in which the pH was as low as 1.5 (see additional file 1).

Although *C. sakazakii *is well known for its association with infections of low-birth weight, preterm babies through contaminated PIF, in this study it was isolated from the enteral feeding tubes of two neonates receiving breast milk and ready to feed formula, respectively. *C. sakazakii *was the sole isolate from the breast milk tube, and was co-isolated with *E. cancerogenus *from the ready to feed formula tube. It should be noted that previously a *C. sakazakii *neonatal infection has been associated with breast milk [34]. Clinical isolates of *Cronobacter *can also produce profuse capsular material which may contribute to biofilm formation [3]. Non-*Enterobacteriaceae *isolated included *P. fluorescens *and *P. luteola*; see additional file 2. These organisms are well known for their ability to form biofilms which could entrap other organisms less able to colonise the tubing wall. Therefore their presence may enhance multiorganism biofilm formation.

The average *Enterobacteriaceae *count in the tube lumen was 10^7 ^cfu/ml, and the average residual liquid volume was 250 μl. Therefore, the number of *Enterobacteriaceae *present in the lumen was in the range from 10^2 ^to 10^7 ^cfu. This equates to the number of *Enterobacteriaceae *that would have entered the neonatal stomach with the next feed. The organisms probably originate from the attached bacterial biofilm, and therefore a reduction in biofilm formation should lead to lower numbers of organisms ingested via the lumen contents. The presence of the residual liquid was independent of the feeding regime, and reflected general feeding practices. The presence of the liquid was unexpected as normal practice is to flush the tube after feeding with a small volume of air or water to clear it. This residual liquid is a potential risk factor for neonatal infection that could be reduced by changing feeding practices in the NICU. There was limited opportunity for bacterial multiplication in the feed at room temperature during the feeding period (< 30 min) compared with the tube at 37°C which can be in place for > 48 h (see additional file 1). Therefore the enteral feeding tube could be a very important source of bacteria entering neonates, and would act as a significant amplifying step for opportunistic intestinal pathogens. These organisms would have entered the stomach as a bolus with the enhanced acid tolerance enabling them to survive the gastric acid and subsequently greater potential to colonise and infect the neonate.

The microbiological safety of neonatal feeds should not be exclusively focussed on reconstituted PIF due to *C. sakazakii*, but also on the general preparation and practices of enteral feeding to reduce the risk of exposure to other *Enterobacteriaceae *some of which may carry antibiotic resistance factors. Therefore, the practice of prolonged placement of enteral feeding tubes in neonates needs to be considered with respect to the increased risk of exposure to bacterial pathogens.

## Methods

### Preparation and administration of neonatal feeds

Powdered infant formula was reconstituted with sterile cold water at room temperature in a sterile bottle. Ready to feed formula was kept in the original bottle. Expressed breast milk (EBM) was obtained using a sterile expressing kit into sterile plastic pots. Fresh EBM was kept for up to 48 hours in a dedicated fridge at 2-4°C. Any EBM which was not to be used as fresh was frozen for up to 3 months in a dedicated freezer at -20°C. When required EBM was defrosted in the fridge and kept for up to 12 hours after removal from the freezer. The neonates were bolus or continuously fed via a nasogastric feeding tube composed of phthalate free PVC (gauge 3.5). Feeds were administered by pouring into a sterile syringe (without plunger) that was attached to the tube, and allowed to flow into the stomach by gravity. Duration of feeding was less than 30 minutes. Occasionally feeds were given by continuous infusion, and the syringe would then be changed every 4 hours.

### Neonatal intensive care unit infections

During the period of sample collection, there were 38 episodes of neonatal infections in NICU 1 and 13 in NICU 2. In NICU 1, 10 infections were due to *Enterobacteriaceae*; 1 *E. cloacae*, and 2 *K. oxytoca*, 3 *K. pneumoniae*, and 4 *E. coli*. Whereas in NICU 2, 5 infections were due to *Enterobacteriaceae*; 1 *E. coli*, 2 *K. pneumoniae*, and 2 *S. marcescens*. The remaining infections in both units were primarily attributed to coagulase negative staphylococci.

### Microbiological analysis

Nasogastric enteral feeding tubes were collected, without pre-selection, over a period of 11 months by nurses as part of their routine care of neonates in intensive care. The tubes were placed in sterile bags, and refrigerated at 5°C until analysis (max. 24 h). The outside of the tubes were sterilized with isopropyl alcohol. Any residual liquid in the tube lumen was flushed into a pre-weighed sterile Eppendorf tube, and the volume determined by weight difference. Using aseptic techniques, the tubing was cut into 2 cm lengths and except for the gastric 2 cm end, placed in 5 ml sterile saline in a conical test tube. The tubes were vortex mixed for 1 min, and then ultrasonicated at 40 kHz for 5 min at room temperature. The tubes were further vortex mixed for 1 min, and decanted into a sterile test tube. The procedure was repeated, and the combined saline rinses were centrifuged in a benchtop centrifuge (2400 g, 10 min). Afterwards, the supernatant was discarded and the bacterial pellet was resuspended in 1 ml sterile saline. The cell suspension was decimally diluted, and 100 μl volumes were spread on Violet Red Bile Glucose agar (VRBGA) plates (LabM, UK). The plates were incubated at 37°C, for up to 48 hours. *Enterobacteriaceae *colonies (red 1-2 mm diameter, usually surrounded by a reddish zone) were counted and representative colony types were subcultured on Tryptone Soya Agar (TSA) plates (Merck, Germany). Isolates were initially identified using phenotypic profiles with ID32 E (bioMerieux), and confirmed using 16S rDNA gene sequence analysis (Accugenix, Delaware, USA).

### Nucleotide sequence accession numbers

The GenBank accession numbers of the *E. cancerogenus* and *E. hormaechei *isolates sequenced in this study are FM883655 to FM883666.

### Antibiotic sensitivity testing

The susceptibilities of *Enterobacteriaceae *isolates to antimicrobial agents were determined by breakpoint on antibiotic supplemented Iso-Sensitest agar (catalog no. CM0471; Oxoid Ltd.) as according to the British Society for Antimicrobial Chemotherapy protocol [35]. The antibiotics tested were amikacin, gentamicin, amoxicillin, cefotaxime, cefuroxime, ceftazidime, ciprofloxacin, amoxicillin-clavulanic acid, gentamicin, meropenem, piperacillin-tazobactam, and trimethoprim (ADATAB; Mast Diagnostics, Bootle, United Kingdom). ESBL production was detected using the combination disc method as described in Health Protection Agency QSOP 51 [36] using ceftazidime-clavulanic acid, cefotaxime clavulanic acid, and cefpodoxime-clavulanic acid combination discs in comparison to individual-antibiotic ceftazidime, cefotaxime, and cefpodoxime discs according to the manufacturer's instructions (Mast Diagnostics, Bootle, United Kingdom).

### Feeding regime questionnaire

During tube collection, a questionnaire concerning the feeding regime was completed by the attendant nurse. The feeding regime, any addition of a thickening agent, age of neonate, duration the tube had been in place, frequency of feeding and stomach pH prior to last feed were recorded. The amount of information for each tube was limited in order to comply with patient confidentiality. No record of patient clinical condition was recorded. Consequently, the source of each tube was anonymous.

### Electron microscopy

The neonatal enteral feeding tube cells were fixed using 3% gluteraldehyde prepared in a 0.1 M phosphate buffer. The tubes were cut into representative 1 cm lengths and dissected longitudinally to expose the inner surface. The tubes were then washed in phosphate buffer and post fixed in 1% (w/v) osmium tetroxide prepared in 0.1 M phosphate buffer, post fixed in 1% osmium tetroxide, dehydrated through a gradual series of alcohol's up to 100% alcohol and then treated with hexamethyldisilazane for 5 minutes. The air-dried tubes were then attached to aluminium stubs, sputter-coated with gold and examined using a Stereoscan S250 Mark III SEM at 10-20 KV.

### Data analysis

The data were analysed using analysis of variance (ANOVA) (STATISTICA software, Statsoft Inc., 2300 East 14th St, Tulsa, Ok, 74104, USA). Subsequent comparisons between group means were made using Fisher's protected least significant difference (PLSD) *post-hoc *test.

## Authors' contributions

EH, EK, ML and JC-B carried out the experiments, collated and analysed the data. AH and RA undertook the statistical analysis of the data. CS and JG supervised the collection of the enteral feeding tubes. SS liaised with hospital staff and University personnel. SF designed the study, was the principal investigator and supervised the project. SF drafted the manuscript. All authors revised the manuscript, and approved the final manuscript.

## Pre-publication history

The pre-publication history for this paper can be accessed here:

http://www.biomedcentral.com/1471-2334/9/146/prepub

## Supplementary Material

Additional file 1 **Summary of neonates' age and feeding regimes**. Collation of data of the neonates sampled, their feeding regimes and gastric pH values. Click here for file

Additional file 2 **Isolation of *Enterobacteriaceae *from biofilms on nasogastric enteral feeding tubes of neonates receiving a range of feeding regimes**. Identification of *Enterobacteriaceae *isolated from biofilms inside neonatal enteral feeding tubes collated according to the feeding regime of the neonate. Click here for file

Additional file 3 **Antibiogram profile of *Enterobacteriaceae *isolated from neonatal nasogastric enteral feeding tubes**. Antibiotic resistance and sensitivity profiles for the *Enterobacteriaceae *isolated from neonatal enteral feeding tubes. Click here for file
