# Supplementary material for: Neonatal enteral feeding tubes as loci for colonisation by members of the Enterobacteriaceae
Source: BMC Infect Dis. 2009 Sep 1;9:146. doi: 10.1186/1471-2334-9-146 (PMC2749046; doi:10.1186/1471-2334-9-146)
Supplement: Additional file 1 — Summary of neonates' age and feeding regimes. Collation of data of the neonates sampled, their feeding regimes and gastric pH values. [file 1471-2334-9-146-S1.doc]

Additional file 1. Summary of neonates’ age and feeding regimes.

| Feeding regime | Tube in place (h) | | | | | | Gastric pH  (range) | Age (weeks) | | | | | Frequency of feeding | | | | |
| --- | --- | --- | --- | --- | --- | --- | --- | --- | --- | --- | --- | --- | --- | --- | --- | --- | --- |
|  | <6 | 6-11 | 12-17 | 18-23 | 24-48 | >48 |  | <1 | 1-2 | >2-3 | >3-4 | >4 | Cont.^e^ | 2 h | 3 h | 4 h | 3 h by 7^f^ |
| Breast milk only (21)^b^ | 5(25)^d^ | 3(14) | 2(10) | 3(14) | 6(29) | 1(5) | 2.5 (1.5-6) | 9(43) | 2(10) | 6(29) | 2(10) | 2(10) |  | 18(86) | 1(5) | 1(5) |  |
| Fortified breast milk (37) | 9(24) | 5(14) | 7(19) | 4(11) | 6(16) | 5(14) | 4.1 (2-5.5) | 1(3) | 4(11) | 7(19) | 4(11) | 21(57) |  | 23(62) | 11(30) |  | 3(8) |
| Ready to feed formula (26) | 6(23) | 7(27) | 6(23) | 1(4) | 4(15) | 2(8) | 3.5 (2-5.5) | 4(15) | 9(35) | 3(12) |  | 10(39) | 2(8) | 10(39) | 10(39) | 2(8) | 1(4) |
| Reconstituted PIF^c^ (8) |  | 2(25) |  | 3(38) | 2(25) | 1(13) | 4.3 (3.5-5.5) |  | 1(13) |  | 1(13) | 6(75) | 2(25) | 4(50) | 1(13) | 1(13) |  |
| Mixed feeding regime (27) | 4(15) | 5(19) | 2(7) | 8(30) | 3(11) | 5(19) | 3.8 (1.5-5.5) | 1(4) | 5(19) | 4(15) | 4(15) | 13(48) | 4(15) | 14(52) | 8(30) | 1(4) |  |
| Nil by mouth (10) | 4(40) | 2(20) |  | 1(10) |  | 3(30) | 3.5 (2-6) | 3(30) | 2(20) | 2(20) | 1(10) | 2(20) |  |  |  |  |  |
| Total | 28 | 24 | 17 | 20 | 21 | 17 |  | 18 | 23 | 22 | 12 | 54 | 8 | 69 | 31 | 5 | 4 |
| (%) | 22 | 19 | 13 | 16 | 16 | 13 |  | 14 | 18 | 17 | 9 | 42 | 6 | 54 | 24 | 4 | 3 |

a Number of samples analysed per feeding regime

b Not recorded duration of tube in place for one sample.

c PIF = Powdered infant formula

d Numbers in parenthesis are percentage values

e Continuous feeding

f Seven feeds every 3 h over a 24 h period.
