# Supplementary material for: Neonatal enteral feeding tubes as loci for colonisation by members of the Enterobacteriaceae
Source: BMC Infect Dis. 2009 Sep 1;9:146. doi: 10.1186/1471-2334-9-146 (PMC2749046; doi:10.1186/1471-2334-9-146)
Supplement: Additional file 2 — Isolation of Enterobacteriaceae from biofilms on nasogastric enteral feeding tubes of neonates receiving a range of feeding regimes. Identification of Enterobacteriaceae isolated from biofilms inside neonatal enteral feeding tubes collated according to the feeding regime of the neonate. [file 1471-2334-9-146-S2.doc]

Additional file 2. Isolation of *Enterobacteriaceae* from biofilms on nasogastric enteral feeding tubes of neonates receiving a range of feeding regimes.

| Feeding regime (n,%) | Number of samples positive for *Enterobacteriaceae* | *Enterobacteriaceae* species | | | | | | | |  |
| --- | --- | --- | --- | --- | --- | --- | --- | --- | --- | --- |
|  |  | *E. coli* | *E. cancerogenus* | *E. hormaechei* | *K. pneumoniae* | *R. planticola* | *R. terrigena* | *S. liquifaciens* | *S. marcescens* | Others |
| Breast milk (21,16) | 11(52)^a^ | 3(4) | 8(38) | 12(57) | 2(10) |  |  | 2(10) | 7(33) | 1 *C. sakazakii*,  *P. fluorescens*, *Raoultella* spp. |
| Fortified breast milk (37,29) | 29 (78) | 10(27) | 19(51) | 10(27) | 21(58) | 1(3) | 1(3) | 5(14) | 5(14) | 1 *E. vulneris*, *E. amnigenus*, *Raoultella* spp., *Kluyvera* spp., *S. odorifera* |
| Ready to feed formula (26,20) | 21(81) | 7(27) | 8(31) | 4(15) | 7(27) |  | 3(14) | 4(15) | 18(69) | 1 *C. sakazakii*,  *C. freundii,*  *P. luteola*,  *E. amnigenus*, |
| Reconstituted PIF (8,6) | 7(88) | 5(6) | 3(4) |  |  |  | 6(75) | 2(25) | 3(38) | 1 *Y. enterocolitica*, *K. ozaena,*  *C. violaceum* |
| Mixed (27,21) | 21(78) | 1244() | 13(48) | 14(52) | 1(4) | 3(11) | 3(11) | 2(7) | 5(19) | 2 *S. odorifera,*  *1 C. violaceum* |
| Nil by mouth (10,8) | 8(80) |  | 2(20) | 4(40) | 1(10) |  |  |  | 8(80) |  |
| Total 129 (%) | 97 (75) | 37 (29) | 53 (41) | 44 (34) | 32 (25) | 4 (3) | 13 (10) | 15 (12) | 46 (36) |  |

a Numbers in parenthesis are percentage values.
