# Supplementary material for: Neonatal enteral feeding tubes as loci for colonisation by members of the Enterobacteriaceae
Source: BMC Infect Dis. 2009 Sep 1;9:146. doi: 10.1186/1471-2334-9-146 (PMC2749046; doi:10.1186/1471-2334-9-146)
Supplement: Additional file 3 — Antibiogram profile of Enterobacteriaceae isolated from neonatal nasogastric enteral feeding tubes. Antibiotic resistance and sensitivity profiles for the Enterobacteriaceae isolated from neonatal enteral feeding tubes. [file 1471-2334-9-146-S3.doc]

Additinal file 3. Antibiogram profile of *Enterobacteriaceae* isolated from neonatal nasogastric enteral feeding tubes

| Organism (n) | TM | | AML | | AMC | | TAZ | | AMI | | CAZ | | CTX | |
| --- | --- | --- | --- | --- | --- | --- | --- | --- | --- | --- | --- | --- | --- | --- |
|  | S | R | S | R | S | R | S | R | S | R | S | R | S | R |
| *E. coli* (37) | 26(70)^a^ | 11(3) | 7(19) | 30(81) | 27(73) | 10(27) | 34(92) | 3(8) | 37(100) | 0(0) | 33(89) | 4(11) | 33(89) | 4(11) |
| *E. cancerogenus* (53) | 52(98) | 1(2) | 0(0) | 53(100) | 45(85) | 8(15) | 53(100) | 0(0) | 52(98) | 1(2) | 53(100) | 0(0) | 50(94) | 3(6) |
| *E. hormaechei* (44) | 30(68) | 14(22) | 12(27) | 32(73) | 15(34) | 29(66) | 35(80) | 9(20) | 44(100) | 0(0) | 35(80) | 9(20) | 34(77) | 10(23) |
| *K. pneumoniae* (32) | 23(72) | 9(28) | 1(3) | 31(97) | 28(86) | 4(13) | 32(100) | 0(0) | 32(100) | 0(0) | 32(100) | 0(0) | 32(100) | 0(0) |
| *Raoutella* spp. (19) | 19(100) | 0(0) | 5(26) | 14(74) | 18(95) | 1(5) | 19(100) | 0(0) | 19(100) | 0(0) | 19(100) | 0(0) | 19(100) | 0(0) |
| *S. liquefaciens* (15) | 0(0) | 15(100) | 4(27) | 11(73) | 8(53) | 7(47) | 14(93) | 1(7) | 15(100) | 0(0) | 14(93) | 1(7) | 14(93) | 1(7) |
| *S. marcescens* (46) | 12(26) | 34(74) | 0(0) | 46(100) | 0(0) | 46(100) | 46(100) | 0(0) | 46(100) | 0(0) | 46(100) | 0(0) | 46(100) | 0(0) |
| Total (n=246) | 162 | 84 | 29 | 217 | 141 | 105 | 233 | 13 | 245 | 1 | 232 | 14 | 228 | 18 |
| (%) | (66) | (34) | (12) | (88) | (57) | (43) | (95) | (5) | (99) | (1) | (94) | (6) | (93) | (7) |

Footnotes: S= sensitive, R = resistant. TM trimethoprim, AML amoxicillin, AMC amoxicillin-clavulanic acid, TAZ piptazobactam, AMI amikacin, CAZ ceftazidime, CTX cefotaxime.

a Numbers in parenthesis are percentage values.
